# Understanding the Role of Patients and Carers in a Virtual Hospital Through Journey Mapping: *Multi‐Method Triangulation Analysis*

**DOI:** 10.1111/hex.70654

**Published:** 2026-04-05

**Authors:** Kanesha Ward, Tim M. Jackson, Shannon Saad, Sarah J. White, Jenna Bartyn, Sue Amanatidis, Owen Hutchings, Annie Y. S. Lau

**Affiliations:** ^1^ Centre for Health Informatics, Australian Institute of Health Innovation Macquarie University Sydney New South Wales Australia; ^2^ Sydney Local Health District Virtual Hospital (Sydney Virtual ‐ Previously Known as rpavirtual) Sydney Local Health District Sydney New South Wales Australia; ^3^ School of Clinical Medicine ‐ Discipline of General Practice University of New South Wales Sydney New South Wales Australia; ^4^ Faculty of Medicine and Health Sciences Macquarie University Sydney New South Wales Australia

**Keywords:** carer involvement, journey mapping, patient experience, qualitative study, triangulation, virtual care, virtual hospital

## Abstract

**Background:**

As virtual hospital models expand, understanding patient and carer experiences is essential to ensure care remains person‐centred, equitable, accessible and effective. While digital tools can improve access and safety, there remain gaps in understanding the roles of patients and carers. Targeted support from health services and policy makers is needed to enable patients and carers to undertake remote monitoring and self‐management activities.

**Objectives:**

To first identify key roles of patients, carers and healthcare workers within virtual hospital care, Acute Respiratory model of care. Second, to identify bright spots (i.e., positive experiences) and pain points (i.e., challenges) reported by carers to improved experiences.

**Method:**

A multi‐methods study involving a method triangulation analysis of 3282 patient‐reported experience measure surveys and 52 semi‐structured interviews with patients, carers and healthcare workers. Patient and carer insights were mapped across three phases of the virtual hospital journey: admission, treatment and discharge. A content and thematic analysis approach was used to (i) present patient narratives, (ii) identify stakeholder roles and (iii) carer‐specific bright spots and pain points.

**Results:**

Virtual hospital care required patients and carers to take on new *technical and digital support roles*, particularly in onboarding, equipment use and troubleshooting. Existing roles, including logistical, household and clinical support, expanded in scope. This was especially for carers acting as *extensions or substitutes* for patient responsibilities when patients had low digital literacy, language barriers or reduced mobility. Participants valued the convenience, safety and responsiveness of virtual care, but reported that any inconsistencies in communication, technology usability and insufficient support for carers can lead to poor experiences.

**Conclusion:**

Patient and carer experience in virtual care is shaped by how clearly expectations are communicated, how effectively technology is supported and how meaningfully carers are engaged. Mapping their journey provides an empirical summary and future research foundation for improving upon virtual care processes so that they are inclusive, responsive and better aligned with user needs.

**Patient or Public Contribution:**

A patient and carer consumer representative panel contributed to the study design and conceptualisation, informing the development of the interview guides. The panel also provided input on the use of visualisation methods and on plans for dissemination of the findings.

**Trial Registration:** Not applicable.

**Abbreviations**
ED: emergency department
eMR: electronic medical record
HCW: healthcare worker
NSW: New South Wales
NUM: nurse unit manager
PJM: patient journey map(s)/patient journey mapping
PREM: patient‐reported experience measure
REDCap: Research Electronic Data Capture
SLHD: Sydney Local Health District

## 1 Introduction

As virtual hospital models continue to grow in response to changing healthcare needs, understanding the lived experiences of patients and carers becomes increasingly important to ensure these services remain patient‐centred and effective [1]. Virtual hospitals deliver remote hospital‐level clinical care to patients at home supported by digital technology (e.g., remote monitoring technologies, digital communication platforms) and multi‐disciplinary clinical oversight [2]. These models are designed to improve hospital capacity, reduce healthcare costs and allow patients to receive care in familiar home environments while maintaining clinical safety and quality of care [1, 2].

The rapid implementation of virtual hospitals, particularly during periods of health system strain such as the COVID‐19 pandemic, prioritised service scale‐up, operational functionality and clinical safety [3]. As a result, early service development did not always incorporate detailed patient and carer experiential insights [3]. However, as virtual care models continue to evolve, there is increasing recognition that patient and carer experiences are essential to designing services that are acceptable, sustainable and responsive to the realities of receiving and delivering care in the home setting.

Emerging evidence highlights both the benefits and challenges of home‐based care. For example, a recent systematic review synthesised patient and carer perspectives of Hospital‐at‐Home care for older adults, identifying both strengths and challenges that offer actionable insights to refine virtual care models and support home‐based care delivery [4]. These findings emphasise the need for more comprehensive research capturing experiences across the full care journey and including both patient and carer perspectives.

Despite the expansion of virtual hospital services, limited research has examined experiences across the entire care journey, from onboarding to treatment and eventual discharge. Additionally, the perspectives and contributions of carers (e.g., patients' family members, friends, partners, companions, paid carers and other caregivers), whose support is often critical yet under‐recognised, are under‐explored in virtual care delivery. For example, Ramamoorthi et al. used patient journey mapping (PJM) to document patient successes and dissatisfactions with virtual care modalities in mental health, chronic care and surgery contexts [5]. However, their analysis did not address the roles, responsibilities or evolving involvement of patients and carers.

This research addresses these gaps by examining experiences across a virtual hospital Acute Respiratory model of care. It aims to explore evolving roles and responsibilities of patients, carers and healthcare workers (HCWs) across the care journey, identify key pain points and bright spots (particularly from carers' perspectives) and capture both practical and emotional dimensions. For this study, a bright spot is defined as a strength of the experience, something that is working particularly well and consistently valued by stakeholders. A pain point refers to a specific barrier, frustration, challenge or negative aspect of the service experience reported by stakeholders. The findings of this study are intended to generate actionable insights on how to better support patients and carers and inform the future development of virtual care services.

## 2 Methods

### 2.1 Study Design

This study employed PJM to explore the experiences of patients, carers and HCWs within an Acute Respiratory virtual hospital model of care. PJM provides a visual and structured representation of roles, activities, successes, burdens and support needs across the care journey [6, 7]. The approach integrates empirical data triangulated from multiple stakeholder groups. In doing so, the study contributes a unique methodological approach that systematically captures and represents patient and carer roles within virtual models of care.

Data were drawn from participants associated with Sydney Virtual (previously known as **rpa**virtual) in New South Wales (NSW), Australia. Details of the data collection have been published in a protocol study [2].

### 2.2 Study Participants

Participants included patients receiving care from, carers of patients of or HCWs working within the Acute Respiratory model of care. In this study, carers are defined as individuals providing support to patients, including family members, partners, friends, companions and paid carers. Most carers in the dataset were family members or partners; however, other carer types were also represented. Due to the anonymous nature of some dataset components, the carer type could not always be determined.

### 2.3 Setting

The Acute Respiratory model of care offers nursing, medical and allied healthcare for patients with mild to moderate respiratory illness (including community‐acquired pneumonia, COVID‐19, influenza or other viral infection, mild‐moderate infective exacerbation of chronic obstructive pulmonary disease or asthma or exacerbation of other chronic lung diseases).

Patients are referred to Sydney Virtual either by the emergency department (ED), inpatient specialist team or their community general practitioner (GP). Upon entering the service, patients are provided with a supply of medication and the Sydney Virtual remote wearables devices pack (including an oximeter, thermometer and patient information). The model of care includes 24‐h nursing support through a 24‐h phone call line, daily virtual medical and/or nursing reviews by phone or video call, 24‐h email messaging and other care or monitoring tasks as required.

Over the course of 4 years (2020–2024), the model of care at Sydney Virtual has evolved significantly in response to service maturation and patient experience feedback. Changes included reducing routine call frequency from three calls daily to one to two calls based on patient need, the introduction of a Digital Patient Navigator role in 2021 to support patients with technology concerns, transition from Zoom to Microsoft Teams in 2023 and the introduction of a remote patient‐facing monitoring application in 2024. The app is prescribed to patients to automatically track and upload wearable device measurements via Bluetooth to the patients' electronic medical record (eMR). Given limited exposure to the patient‐facing application during the study period, findings of this study likely reflect overall service maturation rather than effects of the application alone.

### 2.4 Data Collection

A multi‐methods approach, triangulating findings from the following:

i. Qualitative semi‐structured interviews with patients, carers and HCWs. Participating HCWs included nurses, medical clinicians/doctors, nurse unit managers, physiotherapists and IT/logistical support staff.
ii. Patient‐reported experience measure (PREM) surveys collected between March 2020 and August 2024.
iii. Preliminary observations of virtual hospital consultation calls.

Integrating these sources enabled a multi‐perspective understanding of patient and carer experiences, validating findings across methods [8]. HCW perspectives were primarily to contextualise and interpret patient and carer experiences.

#### 2.4.1 Recruitment

Voluntary sampling was used. Information about the study was shared with HCWs via recruitment emails, posters and educational sessions delivered by the research team. HCWs passed onto patients the study advertisement material and sought permission during routine care to share their contact details to the MQ team to minimise ethical risks associated with contacting individuals during acute illness or treatment. Patients were encouraged to ask their carers to participate in the research study. The MQ team then sought informed consent. HCWs were not involved in informed consent elicitation processes. If they consented, the REDCap (Research Electronic Data Capture) e‐consent form was forwarded to those participants to obtain signed consent to participate in observations and interviews. In addition, early in the study, retrospective recruitment was conducted via SMS outreach to a cohort of discharged patients. PREM survey data was collected by Sydney Virtual as part of standard care and provided for analysis. Participants were informed that their decision to participate (or not) in the research would not affect the treatment they received or their employment status at Sydney Virtual, nor their relationship with MQ.

Due to ethical requirements, direct purposive sampling of patients and carers by researchers was not feasible. However, efforts to capture diverse experiences were supported through broad recruitment across clinical teams, education sessions encouraging participation from varied patient groups and inclusion of multiple stakeholder perspectives.

Interviewers were academic researchers of Macquarie University who were not involved in the participants' medical care or responsible for providing clinical care to patients of Sydney Virtual. Ethics approval was granted by Macquarie University and Sydney Local Health District (SLHD) Human Research Ethics Committees. All participants provided informed consent prior to participation (see Section Ethics Approvals).

#### 2.4.2 Preliminary Observations

Three preliminary observations of virtual consultation calls were conducted early in the qualitative phase of the study with consenting patients and HCWs. These were undertaken to provide external academic researchers with contextual understanding of virtual care delivery workflows and patient–HCW interaction patterns. The researchers joined the calls to passively observe typical virtual hospital interactions.

#### 2.4.3 Interviews

The interview guide was developed collaboratively by a multi‐disciplinary research team, including academic researchers, clinicians, service managers and consumer representatives. The interview guide was pilot tested with a small sub‐set of HCWs and members of the public to ensure clarity and relevance.

During the semi‐structured interviews, all participants were asked to reflect on both positive and negative aspects of the care experience, including elements they believed significantly impacted carers' experiences.

Interviews were approximately 30–40 min in length and led by researchers K.W. and T.M.J. Interviews were conducted between February 2024 and September 2025. All interviews were conducted virtually via Microsoft Teams or via a telephone call. Patients and carers were predominantly interviewed separately unless requested to be interviewed together, which was opted for by some participants. Patients and carers were remunerated $40 (AUD) for their interview participation.

#### 2.4.4 PREM Surveys

The PREM survey is distributed to all patients who receive care on discharge via the REDCap Data Management Software. Several survey domains are covered in the survey including technology use, access to care, overall satisfaction, information and education, trust and confidence, coordination of care and willingness to recommend the service [9]. For the purpose of this study, we focused on the free‐text questions: *What was the best part of the care you received from Sydney Virtual?* and *What part of your care provided Sydney Virtual most needs improving?* PREMs were collected by Sydney Virtual between March 2020 and August 2024. There were 20,392 patient discharges between the period of 1 March 2020 and 31 August 2024. In total, 3352 PREM surveys were completed during this period, indicating a response rate of 16.44%.

### 2.5 Data Analysis and Mapping Procedure

Interview transcripts, preliminary observations of virtual consultations and PREM survey free‐text responses were subjected to a reflexive thematic analysis following Braun and Clarke's 6‐step framework [10]. NVivo 14 (Lumivero) software supported the inductive–deductive coding process. Deductive codes were based on known patient journey phases guided by literature and clinical standards of practice [11], while inductive codes emerged from participants' reported experiences. The research team acknowledge that their professional backgrounds in health research and virtual care may have shaped the interpretation of patient and carer experiences. To enhance reflexivity, multiple researchers were involved in the coding and theme development, and regular discussions were held to examine assumptions, challenge interpretations and ensure that findings remained grounded in the data.

Findings were synthesised into multiple outputs for a comprehensive understanding of experiences: (i) a core journey map describing patient progression through admission, treatment and discharge; (ii) stakeholder‐specific maps of the roles of patients, carers and HCWs; (iii) a tabular summary of roles and activities across phases; (iv) a carer‐focused map of bright spots (i.e., positive elements) and pain points (i.e., challenges), and (v) eight case‐based patient narratives illustrating diverse patient and carer needs. Stakeholder‐specific maps illustrate the different ways stakeholders engage with specific care processes and tasks (i.e., their roles). Bright spots and pain points were identified from carer‐reported experiences only and mapped to the care activities carers described undertaking.

The mapping process began with extracting typical care process descriptions from HCWs, followed by integrating patient and carer‐reported roles. Carer roles were then compared across phases to identify bright spots and pain points. Although patients and carers did not directly co‐create the maps, their narratives, experiences and feedback from their virtual hospital journeys informed all stages of the mapping process.

We followed a qualitative methodology for creating stakeholder narratives [12]. Patient narratives (e.g., personas) are reported to be helpful in illustrating patients' life stories or experiences and in contextualising patient‐reported measures data [13]. These narratives are based on a sub‐set of actual participants. Each narrative describes participant context, including environment (e.g., carer presence and living situation), employment status, self‐assessed digital literacy, a description of their experience and a supporting quote. The participant narratives were used to illustrate how individual factors influence experiences of virtual hospital care and to provide contextual examples of key characteristics across stakeholder cohorts. These included factors such as digital literacy, language, reliance on carers and care complexity, which may shape participant experiences and influence the evolving roles required in virtual hospital care delivery.

A prior scoping review identified mechanisms of patient experience, which were used to guide the classification of bright spots and pain points as indicators of where experiences are functioning well or require improvement [14]. At the same time, a secondary analysis of in‐person GP–patient consultations, conducted by contributing authors of this study, provided a comparative reference point for understanding in‐person care experiences [15]. Bright spots and pain points were coded deductively from the free‐text PREM survey questions.

The data analysis process continued until there were no new codes or themes emerging from the data, and the coding became repetitive [16, 17]. Once thematic saturation was achieved, Lucidchart was used to create diagrams [18]. Maps created by authors were validated with Sydney Virtual clinical HCWs, clinical directors and the research team in dedicated meetings to ensure they reflected real clinical practice.

## 3 Results

### 3.1 Overview of the Virtual Hospital Journey

**Figure 1:**
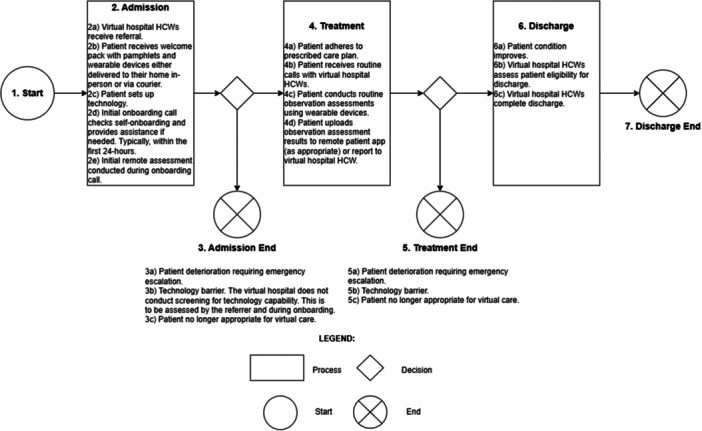
Phases and activities of the Sydney Virtual journey as an Acute Respiratory model of care patient.

Figure 1 visualises a typical patient journey in the Sydney Virtual Acute Respiratory model of care. This journey is structured into three distinct phases: admission, treatment and discharge.

### 3.2 Illustrative Narratives

**Figure 2:**
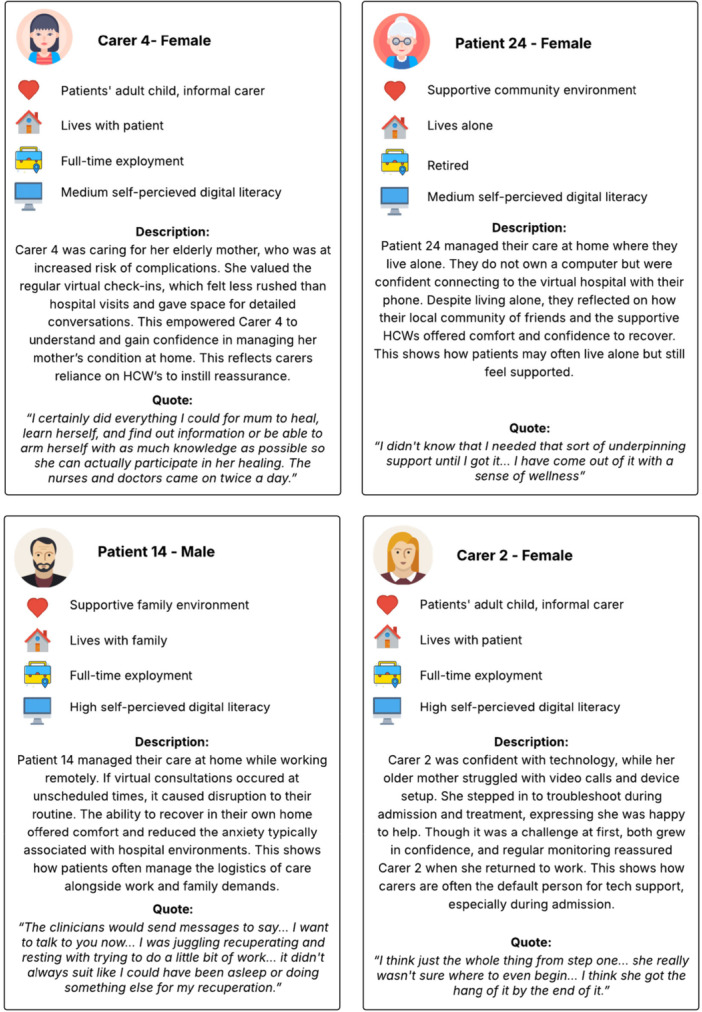
Patient and carer participant narratives.

**Figure 3:**
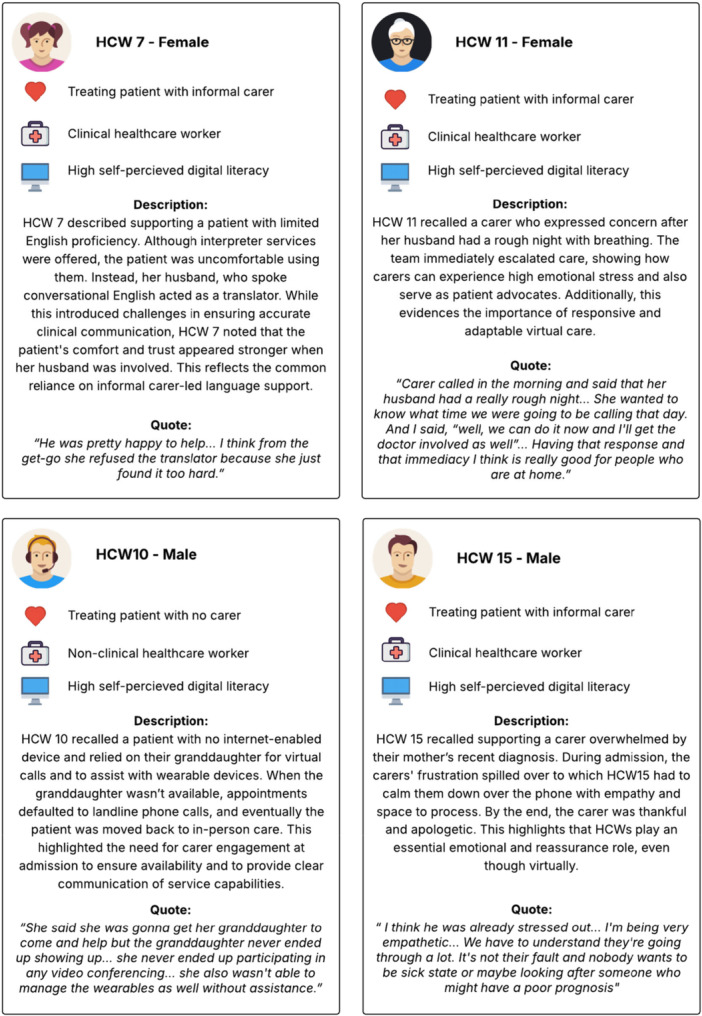
Healthcare worker participant narratives.

To complement the mapped insights, the following eight narratives illustrate the lived experiences of a few carers, patients and HCWs across their virtual hospital journey as reported in the semi‐structured interviews (see Figures 2 and 3). These narratives reflect distinct support needs and barriers, such as digital literacy, language proficiency, reliance on carers and care complexity. Digital literacy was self‐reported by interview participants and interpreted qualitatively during analysis. Levels were described using general categories (low, medium and high) based on participant descriptions of confidence using digital devices and applications. This was not a formal or validated measurement.

### 3.3 Evolving Roles of Patients and Carers

Across the care journey, we identified 11 categories to group roles that patients, carers and HCWs take on, which are categorised using the icon legend (see Table 1). Table 2 outlines the roles of patients, carers and HCWS in Sydney Virtual's Acute Respiratory model of care, respectively. Findings were triangulated across interviews (*n* = 52), PREM free‐text responses (*n* = 3282) and preliminary observations (*n* = 3). PREM responses provided broader population‐level confirmation of themes identified in interviews, while observations provided contextual understanding of how virtual interactions occurred in practice. Consistent themes were observed across data sources, including the importance of clear communication during onboarding, the reliance on carers for technology and monitoring support for some patients and variability in digital confidence influencing care experience.

**Table 1:** Icon legend for Figure 4.

#	Icon	Role category	Description	Stakeholder*
**1**	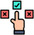	Care advocacy	Voicing needs, navigating consent and patient advocacy	Patient, carer
**2**	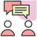	Information sharing	Communicating during encounters, reading through the welcome pack	Patient, carer and HCW
**3**	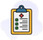	Administrative roles	Assistance with paperwork required for medical discussions (e.g., work letters)	Patient, carer
**4**	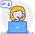	Technology and digital support	Ongoing technical troubleshooting and setup roles	Patient, carer and HCW
**5a**	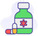	Clinical self‐management	Engaging to caring for self (e.g., journaling logs, medication adherence)	Patient
**5b**	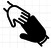	Clinical and care assistance	Assisting with medication management, mobility assistance and assisting with wearable measurements	Carer
**5c**	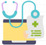	Clinical care and oversight	Clinical instruction and guidance (e.g., prescription of medication, how to conduct wearable measurements and education of ‘red flag’ symptoms)	HCW
**6**	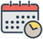	Logistical support	Assisting with system‐facing tasks (e.g., travel assistance, coordinating delivery or pick‐up of wearable devices)	Patient, carer and HCW
**7**	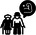	Household support	Assisting with domestic tasks (e.g., cooking, cleaning)	Carer
**8**	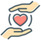	Emotional and motivational support	Role of offering encouragement, reassurance	Carer, HCW
**9**	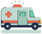	Escalation and risk management	Role as an emergency contact, use of escalation phone lines	HCW

*Note:* *Stakeholders may play a primary or secondary role. Responsibilities are determined by patient factors (e.g., health literacy, digital literacy, family and cultural context, language barriers and clinical condition).

**Table 2:** Roles of patients, carers and healthcare workers throughout the patient journey in Sydney Virtuals' Acute Respiratory model of care.

Role category	Stakeholder	Roles*	Examples
Care advocacy	Patient	a. Provide consent at multiple points (e.g., ongoing care, assessments and escalation) b. Participate in contingency planning (e.g., ‘what if X happens’) c. Act as decision‐maker and self‐advocate, particularly in times of deterioration d. Provide cultural/familial input, particularly for CALD patients e. Ask clarifying questions or challenge decisions/care when unsure	‘There was a little clarification I wanted. So, after I got discharged, I had my discharge papers and then they had the doctor's notes… just to know that you guys were on the same page‐ that if the doctor knew I was gonna take this… as a united front.’—Participant Patient 16 ‘I prefer to use my iPad. So, I asked them to send me a link, and they did that, and I was able to connect through my iPad, which was just a little bit easier for me to use on my phone.’—Participant Patient 25 ‘I was able to phone up and asked questions when needed. Which I did.’—Participant PREM ID 2825, Patient
	Carer	a. Act as a decision‐support (‘third eye’) and escalate concerns when needed b. Next‐of‐kin consent provider in emergencies c. Advocate for patient voice and needs d. Ask clarifying questions or challenge care decisions on behalf of the patient	‘I didn't even think I needed an ambulance. But my daughter said I'm taking you to the emergency.’—Participant Patient 11 ‘I had to call the ambulance and organise all that.’—Participant Carer 3
Information sharing	Patient	a. Report past medical/family history and current symptoms b. Engage in thorough discussions during calls c. Build rapport with HCWs through communication and shared learning for more complete remote assessments	‘Discussion with nursing staff and the doctor calling as soon as they could at all hours of the day as required. That was amazing.’—Participant PREM ID 2802, Patient ‘I had a great time to talk to them… any of my questions, any of my requests as well and they actually took the time.’—Participant Patient 16
	Carer	a. Provide past reports or medication summaries (‘administrative historian’ role) b. Communicate on behalf of fatigued or cognitively impaired patients c. Translate medical information into the patient's preferred language or understanding, particularly for CALD patients d. Build rapport with virtual hospital HCWs to strengthen trust and communication for patient e. Share symptom tracking updates and journaling logs (e.g., dosages, breathlessness, pain and sleep)	‘When he couldn't answer properly anymore, I had to speak for him and explain his symptoms.’—Participant Carer 6 ‘It was more I was listening, and they were checking if there was someone else there and I would say “yes, I'm here and I'm listening to what's what we need to do”. I was kind of second pair of ears really.’—Participant Carer 1 ‘I could record [oxygen and temperature] every three hours for him, rather than him having to do it.’—Participant Carer 1
	HCW	a. Ongoing communication with patients and carers, including updates and education	‘Assurances and tips and guidance what to do ‐ and also what red flags to look for.’—Participant PREM ID 2115, Carer ‘Just being quite clear, being very patient as well… You shouldn't just assume that like it's a clear line and they can hear you well. So, just kind of checking, you know, like “can you hear me ok can you see me ok”.’—Participant HCW 4
Administrative roles	Patient	a. Provide past reports or medication summaries (‘administrative historian’ role) b. Assist with paperwork required for history‐taking discussions c. Confirm accuracy of discharge information for work, insurance or personal records d. Apply for short‐term sick leave, Centrelink/Medicare/Welfare updates or insurance payments	‘A medical form just to keep people out of work without having to ask for it would be nice. So, making the assessment and this is your medical form delivered to your employee.’—Participant Patient 27 ‘I did get a doctor's notice for my lectures.’—Participant Patient 16
	Carer	a. Assist with paperwork for admission, history‐taking or discharge discussions b. Confirm accuracy of discharge information and assist with requesting documentation for work/insurance/personal records (if required)	‘We started to get welcome emails.’—Participant Carer 4 ‘(The patient) just sent me an SMS… then we got some discharge paperwork in the mail.’—Participant Carer 3
	HCW	a. Complete referral onboarding documentation b. Complete eMR treatment documentation c. Complete discharge documentation	‘I talked through with the patients how to use a pulse oximeter and what it does, what it doesn't do… And then I'd add that as a note, in the electronic medical record, just so the nurses were aware that they had these questions.’—Participant HCW 8 ‘I usually start my shift by checking the dashboard to see what patients have been assigned to us today and what cohorts we're responsible for.’—Participant HCW 14
Technology and digital support	Patient	a. Perform ‘technical user’ tasks (i.e., app log‐in, Bluetooth connection of wearable devices, troubleshooting, managing passwords and internet connectivity)* b. Read and act on welcome pack information that requests self‐onboarding of technology* c. Record and upload measurements via app, report to virtual hospital HCWs or use symptoms logs for ad hoc observation assessments*	‘I already had downloaded the app and set myself up… it just reminded me when to put things in.’—Participant Patient 25 ‘There was a text feature, and I was telling her I could not really hear her and then I think she tried some things from her end which did not work. I went off and I joined the link again. And then it started working.’—Participant Patient 16
	Carer	a. Act as ‘technical liaison’ when technically savvy (i.e., log‐in, app download, Bluetooth connection of wearables, troubleshooting, account creation, managing passwords and internet connectivity)* b. Assist with wearable measurements* c. Read and explain welcome pack materials to assist with self‐onboarding (if required)*	‘We had a lot of that case next of kin engaged in their care…So there are few times like you know for a next of kin who they are helping with technical problem.’—Participant HCW 5 ‘We were relying on their granddaughter to be able to be present, to one assist the patient with their wearables, and two to actually set up the teams conferencing.’—Participant HCW 10
	HCW	a. Technical guidance/IT support* b. Initiate remote patient app onboarding (e.g., setup, account creation)* c. Check patient self‐onboarding with wearable devices and providing assistance*	‘I think a lot of staff are very comfortable with using technology and troubleshooting now, and we do have a good network of people here who support each other if they are having any issues, technical issues.’—Participant HCW 2 ‘As part of that initial assessment, we need to play that technical role in setting them up to make sure that moving forward, that's all prepared and that they will be suitable.’—Participant HCW 6
Clinical self‐management	Patient	a. Act as ‘pseudo‐nurses’ (i.e., gather baseline data, track and report observations)* b. Conduct medication management (i.e., administer medications, collect prescriptions, pharmacy coordination and payment/travel) c. Participate in care plans (e.g., exercises, adequate rest and adherence to prescribed plan) d. Request resources (e.g., prescription refills, symptom management advice pamphlets) e. Use journalling logs and trackers (e.g., dosages, sleep, pain and breathlessness) f. Medication reconciliation preparation for discharge (e.g., medication cessation or ongoing prescriptions)	‘Once they know how to put it on, they're all set, and sometimes when you ring, they say, “oh, yeah, I just took it. These are the numbers.” They're proudly one step ahead of you.’—Participant HCW 11 ‘I did it (wearable measurements) a number of times a day… I thought well, I'll see how it's going.’—Participant Patient 17
Clinical and care assistance	Carer	a. Provide ‘pseudo‐nurse’ support (i.e., administering medications, medication records, collecting prescriptions, pharmacy coordination and payment/travel)* b. Support physical care (e.g., mobility, exercise demonstrations and assisting with showers) c. Request resources (e.g., prescription refills, symptom management advice pamphlets)	‘I had to wake him up to make sure he had his inhalation.’—Participant Carer 1 ‘My wife would assist in taking the observations. She found it very helpful because she would ask questions about how best to look after me.’—Participant Patient 12 ‘I actually took time off work… so there definitely needs to be a support person at home… even taking your temperature and the oximeter sometimes is such a challenge.’—Participant Carer 4 ‘Medication script sent through phone instantly so my family could pick up script and use medication straight away that relived my system that stopped me going to hospital.’—Participant PREM ID 2542, Patient
Clinical care oversight	HCW	a. Clinical guidance and oversight b. Patient triage through tiered levels of care c. Medication prescription and oversight d. Review of remote observation/assessment results* e. Conduct discharge assessments and eligibility	‘Knowing that I could leave and go to work and not be concerned or worried that she'd be at home and something might happen… Knowing that she had the ability to just call somebody and that they were checking in with her anyway.’—Participant Carer 2 ‘Tailored health advice for my child.’—Participant PREM ID 3430, Carer ‘One day I uploaded a pulse oximeter that was less than 95%… and they called me within an hour and said, “wait, what's going on?”’—Participant Patient 25 ‘She gave me some information on what can happen with COVID, but also information on the various masks that you can use. She also sent some‐ documents about rpavirtual.’—Participant Patient 3 ‘…they told me at discharge that I should see my GP in two weeks to have some blood tests rechecked.’—Participant Patient 25
Logistical support	Patient	a. Schedule and coordinate with hospital in the home staff (i.e., nurses that visit patients' home) or interpreter services (i.e., CALD support)* b. Manage call schedules and follow‐ups as needed* c. Prepare the home for virtual hospital demands (e.g., medical and household supplies, layout changes to rooms to suit video calls, ensuring privacy, lighting and acoustics)* d. Provide GP/other community care details for discharge planning e. Enable wearable device to return during discharge*	‘I'm a student. So that's very hectic, so being able to do the healthcare virtually and in my own flexible time, that was a big plus point for me.’—Participant Patient 16 ‘At first, I would go into a room on my own and shut the door just for peace and quiet. But then as my family got more used to it, I would just sit here in the lounge room and do it. And it was no issues.’—Participant Patient 20 ‘We know once the patients' discharged, we'll contact the patient to say, “look, you know we're coming back to pick up the device, we're going to be in your area on this particular day. Please text back and this is how you put the device back and leave it out or wait for the driver to contact you.”’—Participant HCW 9
	Carer	a. Assist with coordinating with hospital in the home services (i.e., nurses visiting the patients' home) or interpreter services (i.e., CALD support)* b. Provide travel assistance to pharmacies, investigations, in‐person tests and patients' home c. Financial assistance if the patient is unable to work d. Provide GP/community care details for discharge planning e. Support scheduling and reminders for virtual hospital calls*	‘Going to the chemist, going to do the shopping… it was easier for me to be there.’—Participant Carer 3 ‘…they said drive to this place and pick it up and pay for it. I don't drive… my sister had to collect it from another chemist…if I didn't have her, I probably would have had to catch a taxi.’—Participant Patient 15 ‘I had to make sure the carers I hired had the right insurance, ABN and everything… I was managing all of that, coordinating who came and went.’—Participant Carer 6
	HCW	a. Integrate multi‐disciplinary virtual hospital teams (e.g., IT, logistics and clinical staff) b. Delivery of wearable devices*	‘So, we are always you know let them know they can call us back anytime. And if they call in… we can jump on a video call, we'll do a quick assessment… We'll get the doctor, the nurse, and the patient on and do it all together.’—Participant HCW 4 ‘Medication & health device received within 24 hours after consultation.’—Participant PREM ID 2534, Carer
Household support	Carer	a. Assist in preparing the home for virtual hospital requirements (e.g., gathering supplies, layout, lighting, acoustics and ensuring privacy)* b. Household assistance (e.g., cooking, cleaning and childcare) c. Manage their own care responsibilities if also a patient or recovering, for others in the household (e.g., children)	‘Just making sure that he was OK bringing things to him because it was quite hard for him to move around. He's a pretty breathless… probably doing more my share of the housekeeping and the cooking and other activities.’—Participant Carer 1 ‘They often ring when they're going grocery shopping and ask me if I want anything… always offer to help.’—Participant Patient 17
Emotional and motivational support	Carer	a. Offer motivational encouragement and informal coaching b. Provide emotional support and reassurance during treatment c. Act as advocate when further guidance is required	‘I think it (carer presence) really showed him that someone else was there. I think it was a pretty frightening experience.’—Participant Carer 1 ‘I certainly did everything I could for mum to heal… learn herself and arm herself with as much knowledge as possible so she can actually participate in her healing.’—Participant Carer 4
	HCW	a. Motivational and emotional support (e.g., rapport‐building conversations, encouragement and reassurance)	‘There was an incidence when I was in distress as my mum was struggling to breath. The doctor and nurse took the time to speak with me over the phone and kept talking to me until I was comfortable enough and not crying. They even comforted myself that my mum was progressing well.’—Participant PREM ID 740, Carer ‘You talk about the weather or, you know, are they well enough to walk outside? “It's lovely and sunny outside”. It's just about building rapport and becoming comfortable with each other before you start asking personal questions about their health.’—Participant HCW 11 ‘It was helpful, and it saved my (carer) panicking about whether things were progressing normally or not.’—Participant Patient 12 ‘The doctors and nurses were very inclusive… some made a point of actually remembering my name and including me in conversations, which was great.’—Participant Carer 4
Escalation and risk management	HCW	a. Escalation support during emergencies (e.g., assisting with calling an ambulance)* b. Escalation oversight (e.g., initiate escalation pathways for patients who are deteriorating) c. Consent management—obtaining and revisiting consent d. Organise welfare checks to patient home as required*	‘Monitoring symptoms and organised ambulance when needed.’—Participant PREM ID 2127, Carer ‘If people require paramedics on site or need to come to the emergency department we discuss with the patient at the first visit how to make that happen and reassure their carers how to do that and what different numbers to call and so on, what red flags to look out for.’—Participant HCW 11 ‘If you are not able to contact them, we'll try the next of kin. If not, we'll contact the GP… and if still no, we escalate to a welfare check.’—Participant HCW 15

*Note:* * indicates new roles resulting from virtual hospital care.

**Figure 4:**
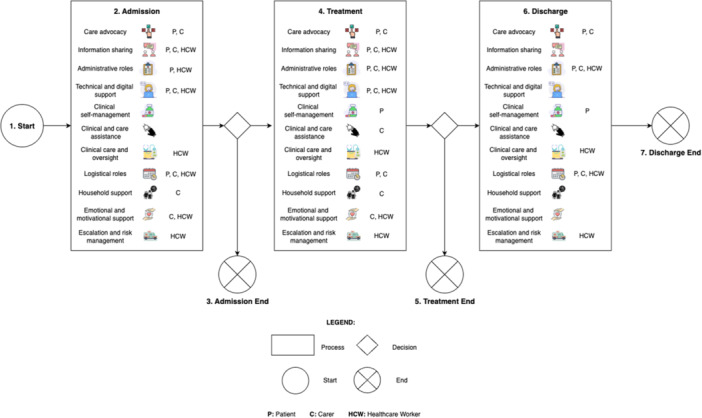
Role of patients, carers and healthcare workers during the virtual hospital journey. Icon legend above.

Figure 4 visually summarises these role categories, highlighting how responsibilities shift and become shared across different phases of virtual hospital care. See Table S1 for the extended Table 2 with additional examples. New roles resulting from virtual hospital caregiving are indicated with an asterisk. *Technical and digital support* roles were entirely new, while *logistical, household, escalation and clinical* roles expanded and evolved with virtual hospital caregiving. Several roles overlap between patients and carers, with carers often stepping in as extensions or substitutes for patient responsibilities when patient capacity is limited (e.g., due to low digital or health literacy, language barriers or reduced mobility).

Overall, patients primarily engaged in clinical self‐monitoring (e.g., symptom reporting) and technology tasks. Carers provided practical and emotional support (e.g., offering reassurance and assisting with domestic tasks) and assisted with monitoring and communication (e.g., assisting with medication). HCWs coordinated clinical assessments, facilitated monitoring and escalation of care. Across phases of care, carer involvement was highest during admission and early treatment, particularly for patients with lower digital literacy/confidence or higher clinical acuity.

### 3.4 Carer‐Specific Bright Spots and Pain Points

Of the 3282 PREM survey responses, 235 were completed by carers on behalf of patients. Of the 235 carer PREMs, 175 carers mentioned one or more bright spots, and 63 carers mentioned one or more pain points (some of whom described multiple spots or points). Each interviewed participant was questioned on the bright spots and pain points they experience, particularly those shared by carers.

**Figure 5:**
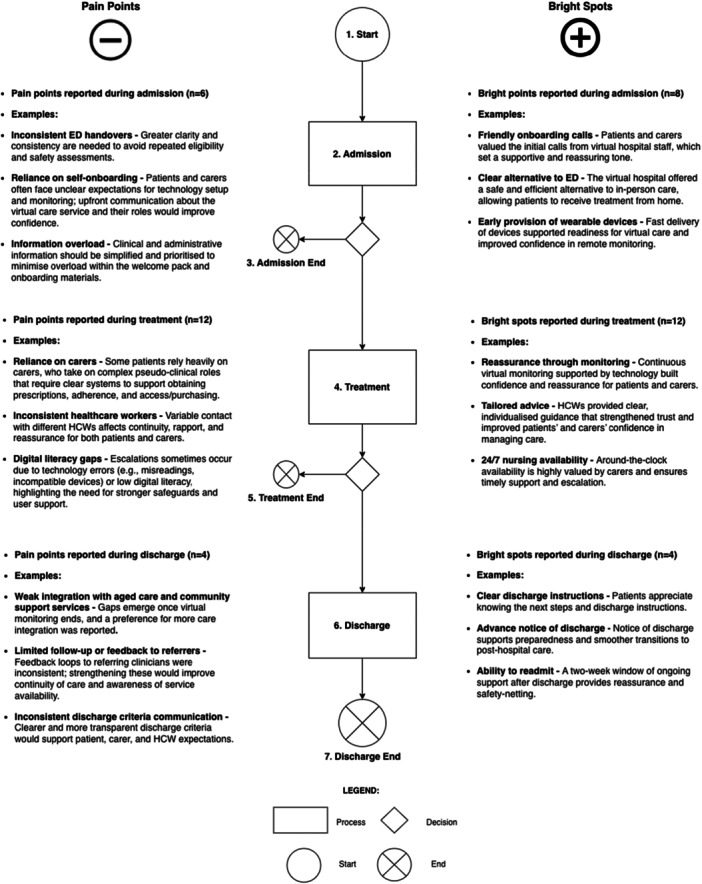
Bright spots and pain points reported by carers of acute respiratory patients within the virtual hospital journey.

Figure 5 presents the phases of the patient journey through the Acute Respiratory model of care, alongside a summary of reported bright spots (e.g., friendly onboarding calls, comfort and empathy) and pain points (e.g., delayed discharge, repetitive information) from carer perspectives. These were informed by the mapped roles and extracted from both PREM surveys and participant interviews, which collectively highlight where carer experiences were most positively or negatively impacted. Insights predominated in the treatment phase. Carers frequently described valuing timely access to clinical information, reassurance and guidance from HCWs, particularly during early stages of acute illness management and periods of clinical uncertainty. This emotional overlay highlights how carers' experiences vary across the patients' journey, offering a visual summary of when and where support is most needed or most effective. See Table S2 for the examples extracted from collected data supporting reporting bright spots and pain points, and an extended version of Figure 5 (see Figure S1).

It is important to note that reported bright spots significantly outweigh reported pain points, as overall experiences were very positive. Additionally, many pain points have been addressed by Sydney Virtual over time as the virtual hospital has evolved and responded to feedback. Pain points are framed as lessons learnt.

## 4 Discussion

### 4.1 Main Findings

This study synthesises critical moments across the virtual hospital journey where patients and carers engage in care‐related activities. Drawing on qualitative insights from interviews, pilot observations and PREM survey data, we found that patients and carers increasingly assume logistical, communicative and relational responsibilities alongside HCWs.

Communication played a prominent role across the care journey and between stakeholders. This leads to a prominent insight of a demand for strong communication skills to explain the ‘why’ behind care processes, troubleshoot technology and navigate expectations. It also leads to a greater observation of using communication to build rapport. As part of the virtual model, patients are required to conduct remote clinical monitoring and physical examinations virtually by HCWs, which is not required of them in traditional in‐person healthcare encounters. This shift places new demands on the physical environment of care (i.e., the home, privacy) and the individuals within it (i.e., available physical support). Carers were often reported to play equally critical but less formally recognised roles, especially for older or dependent patients, where patients required assistance with technology setup, monitoring, advocacy and coordination. These carers appreciated timely access to clinical information, support and guidance.

The integration of carer responsibilities into every phase occurs without system‐level acknowledgement beyond being a ‘next of kin’, which positions carers as an ‘invisible clinical workforce’. Their contributions are often assumed not only by HCWs but also by the patients themselves. HCWs recognise the reliance on carers but highlight systemic gaps in supporting this involvement, especially regarding varying digital literacy, communication or staffing inconsistency and varying health literacy. Our findings show that effective virtual care depends not only on technological skills but also on clear education about expectations, tasks, emotional support for carers and the development of rapport and trust among all stakeholders.

### 4.2 Comparison With Prior Work

PJM is an established method for visualising healthcare experiences across different phases of care delivery, frequently used to identify pain points, emotional burdens and opportunities for service redesign or digital interventions [19, 20, 21]. For example, Meyer [19] demonstrated how longitudinal mapping can highlight unmet needs such as care plan transparency and closed‐loop communication, echoing our study's emphasis on information access and role clarity for both patients and carers. Similarly, Masterson et al. examined the emotional journey of carers of palliative patients, highlighting the burden associated with disease progression, bereavement and post‐loss transitions [20]. Their findings identified the emotional labour involved in caregiving, aligning with our findings. Saragosa et al. conducted an exploratory study using PJM to explore care transitions between hospital and home care [21]. Their study approach mimics our approach of phases, bright spots and pain points, similarly identifying the importance of recognising both patients and carers as central to quality care delivery across the care journey.

While this study demonstrates the usefulness of PJM in mapping patient experiences, few studies have systematically classified the roles of carers throughout the entire care journey or applied PJM to visualising this, especially within virtual models of care. Our research addresses this gap by explicitly involving carers as active participants and charting their emotional, logistical and clinical contributions, as well as their new technical roles across all stages of virtual hospital care. Building on previous advocacy from organisations such as Carers Australia and Carer Gateway, our findings present a task‐oriented taxonomy of carer roles. Consistent with prior literature, carers played key roles in facilitating communication, supporting continuity of care and assisting with navigation of digital systems in virtual care settings. Our findings extend this knowledge by demonstrating how these roles also persist (or may get intensified—e.g., technical setup) when patients are at home during virtual hospitalisation, and their roles evolve across different phases of virtual hospitalisation. We identify roles such as technical liaison (i.e., assist with and troubleshoot technology), advocate and pseudo‐nurse (i.e., healthcare assistants, assist or conduct clinical tasks), many of which carers assume routinely without formal recognition.

A mixed‐methods study by Daddato et al. [22] visualised the emotional paths of carers of spouses with dementia, recognising their roles but mainly concentrating on stress patterns without detailed task‐based responsibilities. In contrast, our maps specify where, when and how carers contribute, particularly in managing virtual hospital care. Our study also highlights how carers enable communication, support clinical continuity after discharge and navigate digital systems. Our findings reinforce previous research that identifies the post‐discharge period as a vulnerable time [23, 24], while also showing how virtual hospital models can extend clinical oversight and reassurance for patients and carers during transitions. By mapping the digital labour undertaken by carers (e.g., setting up wearable devices or interpreting virtual monitoring data), our findings resonate with emerging research on virtual care labour, which notes the reduction of administrative burdens [25] but also sheds light on how clinical responsibilities are externalised to patients and their carers.

### 4.3 Implications for Care

A key implication for policy and practice is the need to redefine the role of carers in healthcare. Many patients can also take on ‘invisible work’ to manage their own care, where digital technology may both support them or create additional considerations for healthcare professionals [26]. In this service model, carer engagement was not formally mandated but occurred organically through clinical processes. In some cases, carers were identified at referral when patients were known to be highly dependent on support. More commonly, carer involvement was identified during initial clinical assessment, where HCWs explored patients' living arrangements and available support. Carers often self‐selected to participate in care activities, including attending virtual consultations, assisting with monitoring tasks and supporting communication with HCWs as a way of providing reassurance and support to patients. Our study highlights the importance of carers' ‘invisible work’, advocating for formal recognition of their role as active contributors to care (e.g., through documentation in patient records, inclusion in care plans and participation in onboarding and escalation processes).

A second implication is clearer preparation for both patients and carers. Clear communication of expectations and clinical tasks is essential to successful virtual care, such as telehealth encounters [27]. At the time of the study, onboarding materials included written fact sheets covering virtual hospital processes, technology setup and troubleshooting, condition‐specific information (e.g., Acute Respiratory pathway), carer support resources (e.g., Carer Gateway information) and, more recently, instructions for remote monitoring applications. These materials were typically provided electronically via email and within the welcome packs with wearable devices. Structured onboarding materials tailored to both patients and carers (e.g., instructional videos, infographics, checklists and troubleshooting guides) may support smoother transitions into virtual care [28]. For example, including training in the use of wearable devices (e.g., oximeters), telehealth platforms, how to's for troubleshooting digital issues and safety netting procedures (i.e., when and how to escalate care). Participants of our study identified that onboarding sessions, such as virtual welcome calls, may also help to clarify roles and build confidence. Additionally, HCWs should receive training in how to engage, support, communicate with and schedule around carers as part of the care team [29].

A third implication relates to the need for emotional support and burden management for patients and carers. Carers frequently experience emotional and cognitive strain [30]. For example, carers may experience moral distress, a concept published in literature as experienced by HCWs, where they experience psychological discomfort or guilt when they perceive a patient has become ‘sicker on their watch’ or when they do not escalate early enough [31, 32]. Carers in this study described experiencing stress related to monitoring patient condition, disruption to sleep and daily routines and balancing employment responsibilities while supporting patients during acute illness phases. While this study did not directly measure carer well‐being outcomes, these findings align with broader literature describing caregiver burden in acute and virtual care contexts [33]. These findings suggest that models of care may benefit from proactively considering carer support needs.

Incorporating carer well‐being checks into routine care, offering access to psychosocial support services or peer networks and embedding simple prompts for HCWs to affirm and support carers may help alleviate this burden and improve rapport [33]. Future research should explore equity and accessibility as not all patients and carers have the same digital, language or cognitive resources. Standardised assessments of digital and health literacy at the point of referral may assist in identifying patients and carers who may need additional support or who may not suit to virtual care [34]. Our findings support research into how virtual hospital services may ensure engagement of digitally literate carers, offer device setup support, multilingual resources, flexible care plans and interpreter services tailored for virtual encounters to address equity concerns [35].

Virtual hospitals should also invest in feedback loops (e.g., post‐discharge carer check‐ins, PREM surveys specific for carers) to validate and improve carer experiences [36]. Carer‐specific feedback tools (e.g., tailored PREM surveys or post‐discharge follow‐ups) may help capture lived experience and drive continuous improvement. The PJMs developed in this study offer a foundation for future co‐design initiatives and quality improvement efforts. These visual tools may be further refined or adapted through use in team huddles, staff onboarding and service design workshops for other virtual care models. Future research should explore the iterative integration of these PJMs into participatory evaluation.

### 4.4 Strengths and Limitations

A key strength of this study is its use of multiple data sources and qualitative methods. This method of triangulation enabled cross‐validation of findings and provided a holistic view of patient and carer experiences across the virtual hospital journey, including under‐explored transition points. Involving multiple researchers in coding and interpretation further enhanced credibility through reflexive discussion. The interview sample size and depth were sufficient to reach thematic saturation [16, 17].

Another strength is the novel application of PJM and participant narratives to synthesise lived experience data across stakeholder groups. This approach enabled integration of multiple data sources into a visually interpretable representation of care experiences and supported identification of how patient and carer roles evolve across different phases of care. In particular, this method made carer contributions more visible within virtual hospital care delivery.

A limitation of this study is that it was conducted in a single model of care in a virtual hospital within an integrated health system, which may limit the transferability of findings to other virtual hospital models of care with different infrastructures, patient populations or staffing models. All eligible patients were invited to participate in the survey and interviews. However, we acknowledge the potential for selection bias as participants may have been more likely to respond if they had particularly positive or negative experiences.

Additional limitations include the small number of preliminary observations, which were intended for contextual understanding rather than primary analytic data; variation in data collection timing across methods, with PREM data collected across multiple years and interviews conducted later, and programmatic changes occurring over the study period that may have influenced participant experiences. Patient and carer interview and PREM data were also limited to individuals who completed the virtual hospital care program as they were collected post‐discharge. Experiences of patients who discontinued care early were primarily captured indirectly through HCW perspectives. As interviews were conducted post‐discharge, participants' recall may have also been affected by the time elapsed or subsequent healthcare experiences.

While efforts were made to include a diverse range of patient and carer perspectives, some subgroups (e.g., non‐English speakers) were under‐represented in our sample compared with the population of patients who received care during the study period. Demographic data were not available for PREM respondents due to the anonymous nature of the survey. Limited demographic and contextual information was available for interview participants; however, due to the small clinical workforce and risk of re‐identification, detailed demographic data for HCWs are not reported.

In this study, PJMs function not only as analytic tools but also as an early draft that stakeholders can help to refine and further validate during co‐design workshops. This approach aligns with implementation science frameworks such as the Consolidated Framework for Implementation Research (CFIR) and the Exploration, Preparation, Implementation, Sustainment (EPIS) framework, which advocate for iterative and exploratory stages prior to full‐scale implementation [37]. Drawing from co‐design frameworks (e.g., an approach employed by Boyd et al. [38]), the journey maps presented here are not intended as final representations but rather empirical foundations to facilitate structured stakeholder engagement. Sanders and Stappers describe such tools as ‘conversation starters’ that facilitate collaborative meaning‐making discussions [39]. However, journey maps in this study were researcher‐led syntheses and have not yet undergone participatory validation by patients and carers. They, therefore, should be considered preliminary and subject to refinement in future co‐design processes. The narratives and maps developed in this study will serve as the foundation for future co‐design studies with patients, carers and HCWs, where they will be refined and adapted through collaborative input.

Although journey mapping approaches are often used iteratively during early program development, this study was conducted after several years of program operation. PREM data were collected retrospectively as part of routine service evaluation, and the interview component was introduced later as part of an academic research collaboration. As such, findings primarily supported reflective service evaluation rather than real‐time iterative redesign. Future work could embed journey mapping prospectively to support continuous service improvement.

## 5 Conclusion

This study highlights the critical role of patients and carers in shaping effective, person‐centred virtual hospital care. Using triangulated journey maps validated by HCWs, we identified key bright spots (e.g., reassurance from clinical oversight, innovative technology and timely communication) and pain points (e.g., challenges with technical troubleshooting, unclear communication of expectations). The evolving roles of carers as coordinators, advocates, pseudo‐nurses and technical liaisons demonstrate the need for more deliberate inclusion of carer perspectives in service design, admission and escalation planning. By making visible both the functional and emotional contributions of carers, our findings extend patient‐centred care frameworks and argue that carers should be treated as core care partners rather than peripheral supporters. Future work should focus on further participatory validation of these visualisations through patients and carers' co‐design studies to build more equitable and sustainable virtual care models.

## Author Contributions

**Kanesha Ward:** conceptualization, writing – original draft, methodology, visualization, writing – review and editing, software, project administration, formal analysis, data curation, investigation. **Tim M. Jackson:** methodology, visualization, writing – review and editing, validation. **Shannon Saad:** methodology, validation, writing – review and editing. **Sarah J. White:** methodology, validation, writing – review and editing, supervision. **Jenna Bartyn:** methodology, validation, writing – review and editing, software, data curation. **Sue Amanatidis:** methodology, validation, writing – review and editing, data curation. **Owen Hutchings:** methodology, validation, writing – review and editing. **Annie Y. S. Lau:** methodology, validation, visualization, writing – review and editing, funding acquisition, supervision, resources.

## Ethics Statement

Ethical approval was granted by the Macquarie University Human Research Ethics Committee for Medical Sciences (reference 520231595552727) and the Sydney Local Health District Ethics Review Committee (Royal Prince Alfred Hospital Zone; reference 2023/ETH01269). The eMR demographics and admission data were de‐identified upon extraction and provided by the virtual hospital. The PREM survey was de‐identified and anonymous upon collection, and the data were provided by the virtual hospital. Patients were informed that participation in the PREMs was voluntary and anonymous. The survey did not elicit sensitive information or identifiable demographic characteristics.

## Consent

All participants provided informed consent prior to participation. Consent was obtained in an electronic, written and or/verbal format.

## Conflicts of Interest

The authors declare no conflicts of interest.

## Permission to Reproduce Material From Other Sources

The authors have nothing to report.

## Supporting information

Supporting Information.

## Data Availability

The data that support the findings of this study are not publicly available due to ethical restrictions and participant confidentiality.
