# Supplementary material for: Understanding the Role of Patients and Carers in a Virtual Hospital Through Journey Mapping: Multi‐Method Triangulation Analysis
Source: Health Expect. 2026 Apr 5;29(2):e70654. doi: 10.1111/hex.70654 (PMC13052216; doi:10.1111/hex.70654)
Supplement: Supplementary file 1 — Supporting Information. [file HEX-29-e70654-s001.docx]

# **Appendix: Supplementary Material (Table S1 and Figure S1)**

**Supplementary Table S1.** Roles of patients, carers, and healthcare workers throughout the patient journey in rpavirtuals’ acute respiratory model of care – Extended table

| **Stakeholder** | **Roles** | **Examples** |
| --- | --- | --- |
| **Patient** | - 1. Care advocacy  1. Provide consent at multiple points (e.g., ongoing care, assessments, escalation) 2. Participate in contingency planning (e.g., “what if X happens”) 3. Act as decision-maker and self-advocate, particularly in times of deterioration 4. Provide cultural/familial input, particularly for CALD patients 5. Ask clarifying questions or challenge decisions/care when unsure | - “I prefer to use my iPad. So, I asked them to send me a link, and they did that, and I was able to connect through my iPad, which was just a little bit easier for me to use on my phone.” – *Participant Patient 25* - “There was a little clarification I wanted. So, after I got discharged, I had my discharge papers and then they had the doctor's notes… just to know that you guys were on the same page- that if the doctor knew I was gonna take this… as a united front.” – *Participant Patient 16* - “I was able to phone up and asked questions when needed. Which I did.” – *Participant PREM ID 2825, Patient* - “I was expressing the view that it might be better for me to stay and have the availability of a bit of oxygen for another 24 hours” - *Participant Patient 12* - “I was thinking of what if one night my pneumonia is really bad, and I have to go to hospital? Am I then back at square one sitting in emergency, waiting for someone? (HCWs said) “OK. Well, actually you're in the system. We can move you through that.”” - *Participant Patient 2* |
|  | - 1. Information sharing  1. Report past medical/family history and current symptoms 2. Engage in thorough discussions during calls 3. Build rapport with HCWs through communication and shared learning for more complete remote assessments | - “I had a great time to talk to them… any of my questions, any of my requests as well and they actually took the time.” – *Participant Patient 16* - “…I spoke to the nurse first and I spoke to the doctor… very nice and very helpful and did explain… everything they were doing” – *Participant Patient 17* - “…they explained everything and I was able to ask questions… it felt like a proper consult, not rushed.” – *Participant Patient 18* - “Discussion with nursing staff and the doctor calling as soon as they could at all hours of the day as required. That was amazing.” *– Participant PREM ID 2802, Patient* |
|  | - 1. Administrative roles  1. Provide past reports or medication summaries (‘administrative historian’ role) 2. Assist with paperwork required for history-taking discussions 3. Confirm accuracy of discharge information for work, insurance, or personal records 4. Apply for short-term sick leave, Centrelink/Medicare/Welfare updates, or insurance payments | - “A medical form just to keep people out of work without having to ask for it would be nice. So, making the assessment and this is your medical form delivered to your employee” – *Participant Patient 27* - “I did get a doctor's notice for my lectures.” – *Participant Patient 16* - “That was a bit intimidating at the beginning because you know, like all these pages to read.” – *Participant Patient 17* |
|  | - 1. Technology and digital support  1. Perform “technical user” tasks (i.e., app log-in, Bluetooth connection of wearable devices, troubleshooting, managing passwords, internet connectivity) 2. Read and act on welcome pack information that requests self-onboarding of technology 3. Record and upload measurements via app, report to virtual hospital HCWs, or use symptoms logs for ad hoc observation assessments | - “I already had downloaded the app and set myself up… it just reminded me when to put things in.” – Participant Patient 25 - “I don't have Wi-Fi. I can use computers at the elementary and I do stuff, but I don't have the computer set up. I use my phone. And so technologically, I thought that was excellent, the way that I was able to just sit back in my chair with my feet up, managing while I did the video calls on my phone... I don't think it's complicated. I just need initial clear instruction and that's it.” - *Participant Patient 24* - “The pulse oximeter was fairly easy to use. The thermometer was actually really easy to use... It communicated with the app seamlessly. I was able to download the app and get myself organised pretty quickly” - *Participant Patient 25* - “There was a text feature, and I was telling her I could not really hear her and then I think she tried some things from her end which did not work. I went off and I joined the link again. And then it started working.” - *Participant Patient 16* |
|  | - 1. Clinical self-management  1. Act as “pseudo nurses” (i.e., gather baseline data, track and report observations) 2. Conduct medication management (i.e., administer medications, collect prescriptions, pharmacy coordination, payment/travel) 3. Participate in care plans (e.g., exercises, adequate rest, adherence to prescribed plan) 4. Request resources (e.g., prescription refills, symptom management advice pamphlets) 5. Use journalling logs and trackers (e.g., dosages, sleep, pain, breathlessness) 6. Medication reconciliation preparation for discharge (e.g., medication cessation or ongoing prescriptions) | - “Once they know how to put it on, they're all set, and sometimes when you ring, they say, ‘oh, yeah, I just took it. These are the numbers.’ They're proudly one step ahead of you” - *Participant HCW 11* - “Some patients write down their observations from when they take them” – *Participant HCW 2* - “If there's something that breaks down, it takes a while to actually get it back on track again. So it takes enormous participation from the patient.” – *Participant Carer 4* - “I did it a number of times a day… I thought well, I’ll see how it’s going.” – *Participant Patient 17* |
|  | - 1. Logistical support  1. Schedule and coordinate with hospital in the home staff (i.e., nurses that visit patients’ home) or interpreter services (i.e., CALD support) 2. Manage call schedules and follow-ups as needed 3. Prepare the home for virtual hospital demands (e.g., medical and household supplies, layout changes to rooms to suit video calls, ensuring privacy, lighting, acoustics) 4. Provide GP/other community care details for discharge planning 5. Enable wearable device return during discharge | - “We know once the patients’ discharged, we'll contact the patient to say, “look, you know we're coming back to pick up the device, we're going to be in your area on this particular day. Please text back and this is how you put the device back and leave it out or wait for the driver to contact you.” – *Participant HCW 9* - “I actually had to write to them and ring them back and say, look, I need a referral today… I need to get in touch with these people today.” – *Participant Patient 24* - “At first, I would go into a room on my own and shut the door just for peace and quiet. But then as my family got more used to it, I would just sit here in the lounge room and do it. And they knew just to be quiet. And it was no issues.” – *Participant Patient 20* - “I wasn't going anywhere. I was super flexible with them like we just go ‘alright, let's do eight o'clock 9:00 o'clock in the morning’. [Carer of Patient 9 name] gets home at 4:30, so after that it was always going to be after then… And one of the times they were really busy. And they said, ‘oh, look, we're short staffed today can we fit you in now?’ And I said yeah let's do it.” – *Participant Patient 9* - “So, I ended up walking home… It was the discharge and the transport home.” – *Participant Patient 26* - “I'm a student. So that's very hectic, so being able to do the healthcare virtually and in my own flexible time, that was a big plus point for me.” – *Participant Patient 16* |
| **Carer** | 1. Care advocacy 2. Act as a decision-support (“third eye”) and escalate concerns when needed 3. Next-of-kin consent provider in emergencies 4. Advocate for patient voice and needs 5. Ask clarifying questions or challenge care decisions on behalf of the patient | - “I didn't even think I needed an ambulance. But my daughter said I'm taking you to the emergency” – *Participant Patient 11* - “Carer called in the morning and said that her husband had a really rough night and had been coughing a lot… She wanted to know what time we were going to be calling that day. And I said, ‘well, we can do it now and I'll get the doctor involved as well’… Having that response and that immediacy I think is really good for people who are at home.” – *Participant HCW 11* - “I had to call the ambulance and organise all that.” – *Participant Carer 3* - *“*The carer expressed frustrated emotions… I had to assist in calming them down by expressing empathy and allowing them space… The patient was very thankful and apologetic.” – Participant HCW 15 |
|  | 1. Information sharing 2. Provide past reports or medication summaries (‘administrative historian’ role) 3. Communicate on behalf of fatigued or cognitively impaired patients 4. Translate medical information into the patient’s preferred language or understanding, particularly for CALD patients 5. Build rapport with virtual hospital HCWs to strengthen trust and communication for patient 6. Share symptom tracking updates and journalling logs (e.g., dosages, breathlessness, pain, sleep) | - “I could record [oxygen and temperature] every three hours for him, rather than him having to do it.” – *Participant Carer 1* - “It was more I was listening, and they were checking if there was someone else there and I would say ‘yes, I'm here and I'm listening to what's what we need to do’. I was kind of second pair of ears really.” – *Participant Carer 1* - “When he couldn’t answer properly anymore, I had to speak for him and explain his symptoms.” – *Participant Carer 6* |
|  | 1. Administrative roles 2. Assist with paperwork for history-taking or discharge discussions 3. Confirm accuracy of discharge information and assist with requesting documentation for work/insurance/personal records (if required) | - “We started to get welcome emails” – *Participant Carer 4* - “(The patient) just sent me an SMS… then we got some discharge paperwork in the mail.” – *Participant Carer 3* |
|  | 1. Technology and digital support 2. Act as “technical liaison” when technically-savvy (i.e., log-in, app download, Bluetooth connection of wearables, troubleshooting, account creation, managing passwords, internet connectivity) 3. Assist with wearable measurements 4. Read and explain welcome pack materials to assist with self-onboarding (if required) | - “We had a lot of that case next of kin engaged in their care…So there are few times like you know for a next of kin who they are helping with technical problem.” - *Participant HCW 5* - “We were relying on their granddaughter to be able to be present, to one assist the patient with their wearables, and two to actually set up the teams conferencing”- *Participant HCW 10* |
|  | 1. Clinical and care assistance 2. Provide “pseudo nurse” support (i.e., administering medications, medication records, collecting prescriptions, pharmacy coordination, payment/travel) 3. Support physical care (e.g., mobility, exercise demonstrations, assisting with showers) 4. Request resources (e.g., prescription refills, symptom management advice pamphlets) | - “My husband helped me with getting prescriptions filled because I needed both antibiotics and the inhaler.” - *Participant Patient 25* - “I had to wake him up to make sure he had his inhalation.” *– Participant Carer 1* - “I actually took time off work… so there definitely needs to be a support person at home… even taking your temperature and the oximeter sometimes is such a challenge” – *Participant Carer 4* - “My wife would assist in taking the observations. She found it very helpful because she would ask questions about how best to look after me” – *Participant Patient 12* - “My mom was, like, send a photo of the inhaler.” – *Participant Patient 16* - “I remind him to check with his GP about certain issues that have come up or to remind him to go and see a specialist… I often attend the specialist appointments with him because I can remember what gets said and I can ask questions that he forgets to ask or remind him about certain things. A bit of a team.… when they're seeing a specialist for the first time, it's a bit- bit overwhelming.” – *Participant Carer 1* - “The simple medical things, even like how to read the oxygen… and having a whole information booklet… really helped and made it less scary.” – *Participant Carer 2* - “I do the measurements… so I’d take his oxygen levels regularly just to make sure… and that was comforting for me.” – Participant Carer 3 - “Medication script send through phone instantly so my family could pick up script and use medication straight away that relived my system that stopped me going to hospital.” – Participant PREM ID 2542, Patient |
|  | 1. Emotional and motivational support 2. Offer motivational encouragement and informal coaching 3. Provide emotional support and reassurance during treatment 4. Act as advocate when further guidance is required | - “I think it (carer presence) really showed him that someone else was there. I think it was a pretty frightening experience.” – *Participant Carer 1* - “I certainly did everything I could for mum to heal… learn herself and arm herself with as much knowledge as possible so she can actually participate in her healing.” - *Participant Carer 4* |
|  | 1. Logistical support 2. Assist with coordinating with hospital in the home services (i.e., nurses visiting the patients’ home) or interpreter services (i.e., CALD support) 3. Provide travel assistance to pharmacies, investigations, in-person tests, patients’ home 4. Financial assistance if the patient is unable to work 5. Provide GP/community care details for discharge planning 6. Support scheduling and reminders for virtual hospital calls | - “Going to the chemist, going to do the shopping… it was easier for me to be there.” – *Participant Carer 3* - “…they said drive to this place and pick it up and pay for it. I don’t drive… my sister had to collect it from another chemist…if I didn’t have her, I probably would have had to catch a taxi.” – Participant Patient 15 - “I had to make sure the carers I hired had the right insurance, ABN and everything… I was managing all of that, coordinating who came and went… I had to stop working to look after him full-time.” – *Participant Carer 6* |
|  | 1. Household support    - - 1. Assist in preparing the home for virtual hospital requirements (e.g., gathering supplies, layout, lighting, acoustics, ensuring privacy)        2. Household assistance (e.g., cooking, cleaning, childcare)        3. Manage their own care responsibilities if also a patient or recovering or others in the houseful (e.g., children) | - “They often ring when they're going grocery shopping and ask me if I want anything… always offer to help.” - *Participant Patient 17* - “Just making sure that he was OK bringing things to him because it was quite hard for him to move around. He's a pretty breathless… probably doing more my share of the housekeeping and the cooking and other activities.” – *Participant Carer 1* - “(Carer) helped me with getting prescriptions filled… getting food from the store, preparing meals.” – *Participant Patient 24* |
| **Virtual hospital healthcare workers** | 1. Clinical care and oversight 2. Clinical guidance and oversight 3. Patient triage through tiered levels of care 4. Medication prescription and oversight 5. Review of remote observation/assessment results 6. Conduct discharge assessments and eligibility | - “The simple medical things, even like how to read the oxygen checker. Having a whole information booklet and everything really helped and made it less scary. I think knowing that her oxygen most of the time is actually really fine and she didn't need to be worried gave me a sense of comfort too. And knowing that I could leave and go to work and not be concerned or worried that she’d be at home and something might happen... Knowing that she had the ability to just call somebody and that they were checking in with her anyway.” – *Participant Carer 2* - “…they told me at discharge that I should see my GP in two weeks to have some blood tests rechecked.” – *Participant Patient 25* - “Discussion with nursing staff and the doctor calling as soon as they could at all hours of the day as required. That was amazing. Knowing I had available support just a phone calls away 24/7. As a carer of a paraplegic husband who had COVID the support gave me a lot more confidence.” - *Participant PREM ID 740, Carer* - “Tailored health advice for my child.” *- Participant PREM ID 3430, Carer* - “The medical information they knew what they were talking about. Very good medical knowledge.”- *Participant PREM ID 2089, Carer* - “One day I uploaded a pulse oximeter that was less than 95%… and they called me within an hour and said, ‘wait, what’s going on?’” – *Participant Patient 25* - “She gave me some information on what can happen with COVID, but also information on the various masks that you can use. She also sent some- documents about rpavirtual.” - *Participant Patient 3* - “Knowing someone was available to contact if I had any issues while isolating - I was not left 'stranded’.” – *Participant PREM ID 165, patient* |
|  | 1. Escalation and risk management 2. Escalation support during emergencies (e.g., assisting with calling an ambulance) 3. Escalation oversight (e.g., initiate escalation pathways for patients who are deteriorating) 4. Consent management – obtaining and revisiting consent 5. Welfare checks to patient home as required | - “Monitoring symptoms and organised ambulance when needed” - *Participant PREM ID 2127, Carer* - “Ability of doctor to visually diagnose patient and make decision to hospitalise patient.” - *Participant PREM ID 2849, Carer* - “If people require paramedics on site or need to come to the emergency department we discuss with the patient at the first visit how to make that happen and reassure their carers how to do that and what different numbers to call and so on, what red flags to look out for.” - *Participant HCW 11* - “’Keep it up, whatever to keep doing what you're doing. But if you ever need us, you know you've got the number you can call at any time’. I do remember that being a frequent reminder that escalation or any questions I could call them.” *- Participant Patient 14* - “If you are not able to contact them, we’ll try the next of kin. If not, we’ll contact the GP… and if still no, we escalate to a welfare check.” – *Participant HCW 15* |
|  | 1. Technology and digital support 2. Technical guidance/IT support 3. Initiating remote patient app onboarding (e.g., set-up, account creation) 4. Checking patient self-onboarding with wearable devices and providing assistance | - “I think a lot of staff are very comfortable with using technology and troubleshooting now, and we do have a good network of people here who support each other if they are having any issues, technical issues.” - *Participant HCW 2* - **“**The technical side of it is we try to troubleshoot it as much as possible. We explain it to them. If not then we have the DPN, the Digital Person Navigator” - *Participant HCW 3* - “Patients are normally pretty cooperative maybe the only barrier would be like, you know, with some patients they may have some technical difficulty, but it's normally easy to troubleshooting” - *Participant HCW 5* - **“**As part of that initial assessment, we need to play that technical role in setting them up to make sure that moving forward, that's all prepared and that they will be suitable.” - *Participant HCW 6* - **“**They helped me through it when I was having trouble in the beginning, and I've never been into technology at all. So, it was just a completely new experience for me.” - *Participant Patient 1* - “I had never had any FaceTime calls or anything. But I had phone call with them to start with and then they told me what to do.” - *Participant Patient 17* |
|  | 1. Logistical support 2. Connecting between multi-disciplinary virtual hospital teams (e.g., IT, logistics, clinical staff) 3. Delivery of wearable devices | - “Medication & health device received within 24 hours after consultation.” - *Participant PREM ID 2534, Carer* - “So, we are always you know let them know they can call us back anytime. And if they call in… we can jump on a video call, we'll do a quick assessment… We'll get the doctor, the nurse, and the patient on and do it all together.” - *Participant HCW 4* - “So, we do get a lot of support from IT team. We ask for a lot of support from that team if we have any issues.” - *Participant HCW 2* |
|  | 1. Administrative roles 2. Completing referral onboarding documentation 3. Completing eMR treatment documentation 4. Completing discharge documentation | - **“**I talked through with the patients how to use a pulse oximeter and what it does, what it doesn't do... And then I’d add that as a note, in EMR or the electronic medical record, just so the nurses were aware that they had these questions” - *Participant HCW 8* - “I usually start my shift by checking the dashboard to see what patients have been assigned to us today and what cohorts we’re responsible for.” – *Participant HCW 14* - “Patients are told they can call us within seven days [post-discharge] and we can readmit patients if they have any additional concerns.”- *Participant HCW 15* |
|  | 1. Information sharing 2. Ongoing communication with patients and carers, including updates and education | - “Assurances and tips and guidance what to do - and also what red flags to look for.” - *Participant PREM ID 2115, Carer* - “Just being quite clear, being very patient as well. That's one of the things that's a little bit different like with virtual care is just reminding yourself to have those pauses and let the other people speak as well, or you know asking them if they have any questions as well, making sure that there's like a good clear connection, you can hear each other ok as well. You shouldn't just assume that like it's a clear line and they can hear you well. So, just kind of checking, you know, like ‘can you hear me ok can you see me ok’” - *Participant HCW 4* - “Sometimes patients can only do video calls in the afternoon, so we adjust… sometimes do a telephone call instead.”- *Participant HCW 15* |
|  | 1. Emotional and relational support 2. Motivational and emotional support (e.g., rapport-building conversations, encouragement, reassurance) | - “There was an incidence when I was in distress as my mum was struggling to breath. The doctor and nurse took the time to speak with me over the phone and kept talking to me until I was comfortable enough and not crying. They even comforted myself that my mum was progressing well. I highly rate them. The most difficult time of our lives and they are amazing people.” - *Participant PREM ID 740, Carer* - “You talk about the weather or, you know, are they well enough to walk outside? “It's lovely and sunny outside”. It's just about building rapport and becoming comfortable with each other before you start asking personal questions about their health.”- *Participant HCW 11* - “It was helpful, and it saved my (carer) panicking about whether things were progressing normally or not.” – P*articipant Patient 12* - “They care for us like family.” – *Participant PREM ID 97, Patient* - “The doctors and nurses were very inclusive… some made a point of actually remembering my name and including me in conversations, which was great.” – Participant Carer 4 |

**Supplementary Table S2.** Extracted examples of carer-specific bright spots and pain points

| **Phase** | **Reported observation** | **Example** |
| --- | --- | --- |
| ***Bright spots*** | | |
| Admission | - Virtual hospital provides a clear alternative to ED - Admission speed has become faster post-COVID-19 - Carers and patients value friendly initial calls from virtual hospital staff - Welcome packs with detailed information pamphlets support onboarding - Eligibility is inclusive and adaptable, meeting the needs of diverse patient groups (e.g., older adults, CALD communities through interpreter services) - Timely access to care and medication - Home-based support is enabled (e.g., carer involvement, hospital-in-the-home services) - Early provision of wearable devices supports monitoring | - “Medication & health device received within 24 hours after consultation” – Participant PREM ID 2534 - “No need goes to ED or call an ambulance before access this service.” – Participant PREM ID 3777 - “RPAvirtual organised to get the antiviral medicine on time. Really appreciated what you did. My experience from rpavirual was more than expected.” – Participant PREM ID 2367 - “It’s sort of like an alternative to being in hospital… That’s better than being there. It’s better to be in your own bed.” – Interview Carer 1 - “…we had a very detailed discussion with the nurse late that afternoon and she went through everything with him about what was going to happen and what he had to do. And that just helped us through the first night…”– Interview Carer 1 - “…the little information booklet… really helped and made it less scary. Knowing her oxygen most of the time is actually really fine… gave me a sense of comfort too.” – Interview Carer 2 - “…we deliver iPads to them and show them how to use it” – Interview HCW 12 |
| Treatment | - Continuous monitoring builds reassurance and confidence - Carers and patients feel that virtual care received is comforting, compassionate, and empathetic - Support is consistent and easily accessible - Carers and patients develop trust in HCWs clinical judgment - Tiered-level of clinical care ensure appropriate escalation - Clinical guidance empowers patients and carers to take on “pseudo-nurse” roles - Innovative technology (e.g., remote patient monitoring apps) enhances monitoring capability - Advice is clear and tailored to individual needs - Receiving care in the home environment encourages deeper discussion due to comfort and privacy - Multi-disciplinary coordination enables effective response times and real-time feedback - Patients’ and carers’ key role in recognising and triggering escalation is acknowledged - 24/7 availability ensures timely support | - “…the telephone number that was repeated constantly by the nurses as to where you can get help. Mum actually wrote it down… it was like a reassurance, I can pick up the phone and I can find someone.” – Interview Carer 4 - “The 1st call nurse, she can speak my language Cantonese, and gave clearly information. she was very patient to answer my question and made me calm.” – Participant PREM ID 2427 - “Ability of Doctor to visually diagnose patient and make decision to hospitalise patient.” – Participant PREM ID 2849 - “Prompt medical attention for a housebound patient” – Participant PREM ID 2684 - “The beautiful nurses always lovely and polite always went the extra mile to help my father. Thank you to all” – Participant PREM ID 3245 - “The regularity of the contact. The unhurried consultations. The insightful questions asked by the team” – Participant PREM ID 3013 - "Able to contact to ask questions” – Participant PREM ID 2981 - “Tips and advice about the red flags what to do” – Participant PREM ID 2350 - “Very supportive and without it would of made me nervous about how my mums illness was going It out my mind at rest as the information was excellent and didn't have to leave the home” – Participant PREM ID 3783 - ““The nurses are really tenacious… they sent a text, phoned mum, sent the link multiple times… She enjoyed talking to a broad spectrum of people… bonded with more mature nurses, but the young ones were enthusiastic.” – Interview Carer 4 - “…their presence reassures patients as well… it’s comprehensive care, we have to not just look after the patients, but also give reassurance to the family as well.” – Interview HCW2 - “…we have level 1 care and level 2 care… Level one care has two nursing reviews per day plus one MO review. Level 2 patients… nursing call two times a day but escalate to MO if concerns.” – Interview HCW 5 - “…HCW assesses patient home environment, seeing how they interact with home space.” – Interview HCW 4 - “…we liaise with medical teams, triage requests for reviews, respond to escalations from nurses and outside services… coordinate across the system.” – Interview HCW 4 - “Discussion with nursing staff and the doctor calling as soon as they could at all hours of the day as required. That was amazing. Knowing I had available support just a phone call away 24/7. As a carer of a paraplegic husband who had COVID the support gave me a lot more confidence.” – Participant PREM ID 2802 |
| Discharge | - Carers and patients value clear discharge instructions - Advance notice of discharge supports preparedness and smoother transitions to post-hospital care - Coordination with GPs strengthens continuity of care - A two-week window of ongoing support after discharge provides reassurance and safety-netting | - “No improvement, only praise. As I'd already been in contact with my Mum's GP, rpavirtual were suggesting treatment as the GP did. So, we were extremely happy with the care given by the Nurse & Doctor we spoke to. Amazing service, & care advice and most of all amazing staff. Thank you very much.” – Participant PREM ID 2036 - “Just reinforcing that “look we are here if you need us” (for discharge)– Interview HCW 2 |
| ***Pain points*** | | |
| Admission | - Clearer and more consistent ED handovers are needed to avoid repeated eligibility assessments - Patients and carers benefit from upfront communication about the virtual care service and their expected roles - Timely initial contact is critical to patient confidence (noting improvements since COVID-19) - Additional strategies are required to support high-need patients without carers (e.g., those with low mobility or cognitive impairment) - Reliable and efficient equipment delivery processes are essential for smooth onboarding - Clinical information should be simplified and better prioritised to reduce information overload within the welcome pack | - “No one explained how or why my son was part of rpavirtual; the first call was totally out of the blue!” – Participant PREM ID 3386 - “Those registration system was confusing.” – Participant PREM ID 1018 - “The initial oxygen reader I had was playing up however the staff were quick to organise another two to come out.” – Participant PREM ID 3310 - “I received several text messages from different departments all wanting to know same information. Could the procedures be a bit more streamlined?” – Participant PREM ID 2725 |
| Treatment | - Medication management requires clearer systems to support carers obtaining prescriptions, adherence, and access/purchasing - Some patients rely heavily on carers for engagement, highlighting the need for alternative support pathways - Tailoring approaches to health literacy is essential for patient and carer success - Over-monitoring can lead to call fatigue, indicating a need to balance reassurance with sustainability - Monitoring capability is valued but consistency in HCWs preferred, which must be balanced against health system capability (i.e., clear expectations of rostering) - Reliability of technology remains a critical factor for patient and staff confidence - Unscheduled communication can disrupt workflow and patient expectations - Repetition of information should be minimised through streamlined communication - Breakdowns in follow-up communication undermine continuity of care - Escalations may occur due to technology errors (e.g., misreading’s, incompatible devices) or digital literacy gaps, requiring safeguards - Carer burden is increased by additional household and emotional tasks, particularly for those travelling (i.e., do not live with the patient) - Limited access to on-site physical support remains a gap for some patients | - “In some calls, the doctor or nurse were requesting the same information which was already provided” – Participant PREM ID 2115 - “At times it was hard with my elderly parents and not living with them ...to have the meetings” – Participant PREM ID 3582 - “Care delivered was focused on my child, but I was also very unwell and because calls were unscheduled, they often woke me when I was finally able to rest/sleep (after caring for my child)” – Participant PREM ID 3430 - “Having the same nurse for the entire period would have been optimal.” – Participant PREM ID 341 - “The doctors and nurses need to review the file notes of previous calls. Important to have good and accurate record management and ensure that a brief summary of call is captured so as not to get the patient to repeat the information each time. This is very important with regards to the list of medication being used by patients.” – Participant PREM ID 2350 - “Sometimes could not understand the nurse. Also found that I was asked questions which were already covered by or advised to other nurses... maybe not communicating information between nurses?” – Participant PREM ID 2130 - “Time to respond to left messages for a call back” – Participant PREM ID 2728 - “The health devices provided didn't work well were inconsistent Due to invalid reading an ambulance was sent to my home” – Participant PREM ID 1611 - “…carers provide the second opinion — like saying, ‘she’s more confused than usual’ or ‘not eating as much’” – Interview HCW 4 - “…technology might stress them… I used to work in IT so I could cope… but if my mother needed this, she’d be freaking out.” – Interview Patient 5 - “…for some patients they’re required to be called three times a day and that’s a lot… sometimes it’s too much for them and the carer, so they say, ‘I’ll take one call a day’.” – Interview HCW 11 |
| Discharge | - Stronger integration with aged care and community support services is needed to sustain care after discharge - Discharge processes can be delayed, highlighting the need for clearer timelines and coordination - Feedback loops to referring clinicians should be strengthened to improve continuity of care - Clearer and more transparent discharge criteria would support patient, carer, and HCW expectations | - “Not having a clear expectation for release” – Participant PREM ID 523 - “Once a patient no longer needs care as decided by their parent a discharge should be organised sooner” – Participant PREM ID 2653 - “People knowing this service is available. I wouldn't have known this service was available… understanding what assistance is available may encourage more carers who require it to access it.” – Participant PREM ID 2802 - “Should have provided aged care referrals as GP not helping with housebound patient who can't get a doctor to see her at home for any checkups” – Participant PREM ID 2684 |

**
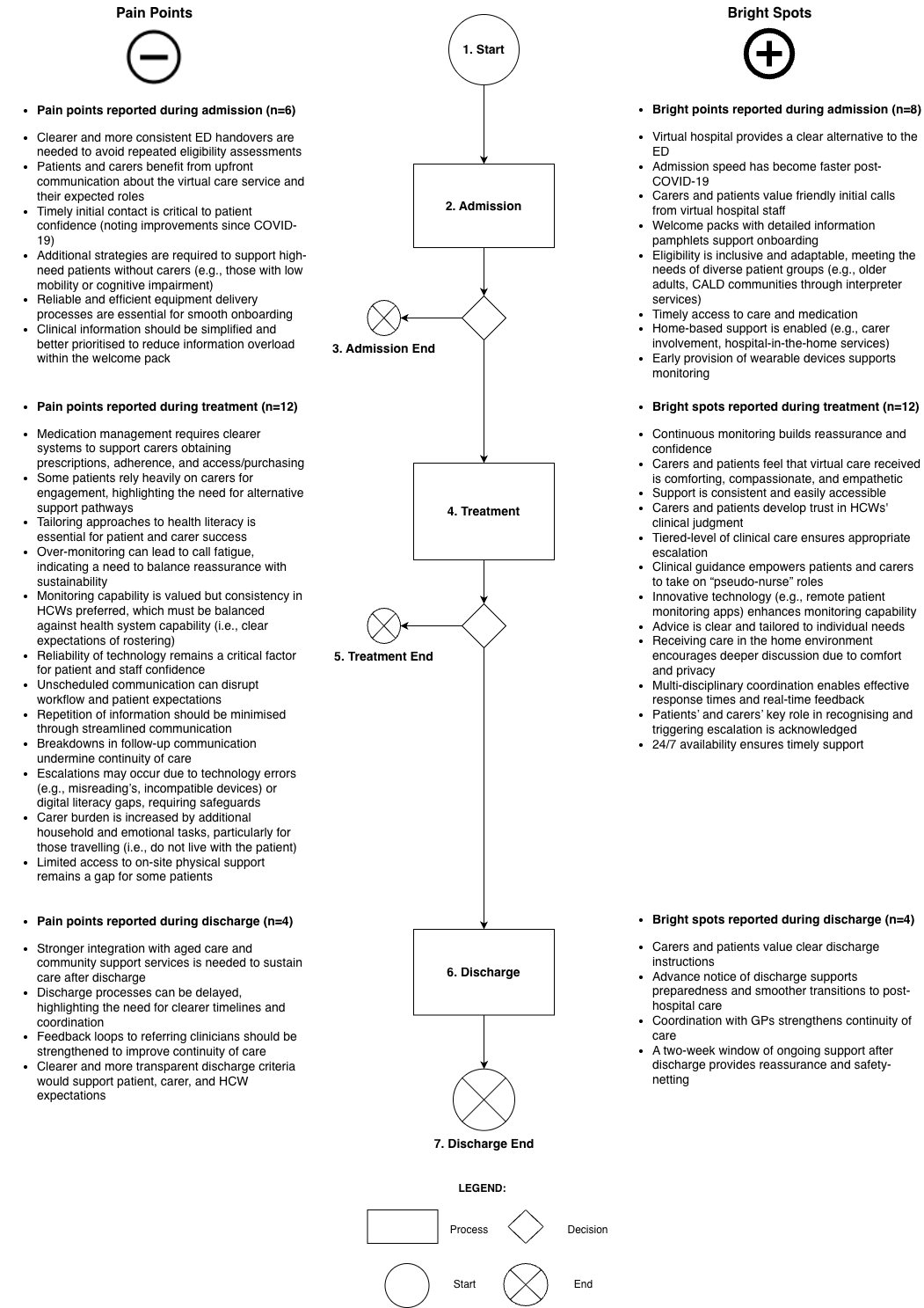
**

**Figure S1.** All bright spots and pain points reported by carers of acute respiratory patients within the virtual hospital journey
